# Associations of the Severity of Dry Eye Symptoms and Signs with Quality of Life in the Dry Eye Assessment and Management (DREAM) Study

**DOI:** 10.3390/vision9010013

**Published:** 2025-02-18

**Authors:** Ellie Cheng, Katherine Han, Yineng Chen, Penny Asbell, Gui-Shuang Ying, the DREAM Research Group

**Affiliations:** 1 School of Medicine, University of Rochester, Rochester, NY 14642, USA; 2 Perelman School of Medicine, University of Pennsylvania, Philadelphia, PA 19104, USA; 3 Center for Preventive Ophthalmology and Biostatistics, Department of Ophthalmology, Perelman School of Medicine at University of Pennsylvania, 51 North 39th Street, Philadelphia, PA 19104, USA; 4 Bioengineering Department, University of Memphis, Memphis, TN 38152, USA; penny.asbell@gmail.com

**Keywords:** dry eye disease, signs, symptoms, quality of life

## Abstract

**Purpose:** To assess associations of the severity of the symptoms and signs of dry eye disease (DED) with the quality of life in patients with moderate-to-severe DED. **Methods:** At baseline, 6 and 12 months, participants (n = 535) were assessed for DED symptoms using the Ocular Surface Disease Index (OSDI) and signs using conjunctival staining, corneal staining, tear break-up time (TBUT), Schirmer’s testing, meibomian gland dysfunction, and tear osmolarity. Quality of life was evaluated using the Short Form Health Survey (SF-36), consisting of a Physical Component Summary (PCS) and Mental Component Summary (MCS). Spearman correlation coefficients (rho) were used to evaluate correlations between the severity of DED and the SF-36. **Results:** At baseline, worse DED symptoms indicated by a higher OSDI total score were correlated with worse PCS (rho = −0.13, *p* = 0.002) and MCS (rho = −0.09, *p* = 0.03) in the SF-36. Worse vision-related function was correlated with a worse PCS score (rho = −0.18, *p* < 0.0001), and worse ocular symptoms were correlated with a worse MCS score (rho = −0.15, *p* < 0.001). More severe DED signs including corneal staining (rho = −0.22, *p* < 0.001), Schirmer test (rho = 0.11, *p* = 0.01), TBUT (rho = 0.14, *p* < 0.001), and tear osmolarity (rho = −0.12, *p* = 0.02) were correlated with a worse PCS score but were not correlated with MCS score (*p* ≥ 0.39). ln longitudinal analysis, only the worsening of ocular symptoms was significantly correlated with the worsening of the MCS score (rho = −0.09, *p* = 0.04). **Conclusions:** In patients with moderate-to-severe DED, there were significant yet weak correlations between the severity of dry eye symptoms/signs and the physical or mental components of the SF-36. Healthcare professionals should offer patients with DED symptom relief and support for the emotional and practical challenges in their daily lives.

## 1. Introduction

Dry eye disease (DED), a multifactorial ocular disorder, affects approximately 9% to 30% of adults in the United States, with higher rates observed among women and increasing age groups [1]. Dry eye disease is characterized by inadequate tear production or excessive evaporation, leading to ocular discomfort and potential damage to the ocular surface [2]. Dry eye manifests through a range of distressing symptoms that can significantly impact an individual’s daily life. Numerous studies have established the profound impact of DED on the quality of life of DED patients. Research has revealed that DED has a direct impact on many aspects of quality of life, including physical health, psychological well-being, and level of independence [3]. For example, Sayegh et al. found that worse ocular discomfort was associated with worse overall DED symptoms and interfered to a greater degree with activities of daily living [4]. Mertzanis et al. found that dry eye disease had a negative impact on everyday life, particularly in daily activities [5].

Patients with DED commonly experience sensations of ocular dryness, grittiness, burning, itching, or foreign body sensation. Additionally, they may experience frequent tearing, fluctuating visual acuity, and sensitivity to light. These symptoms may worsen during prolonged periods of reading, computer usage, or exposure to environmental factors such as wind, smoke, or dry air [3]. Furthermore, inadequate tear production and poor tear quality associated with DED can cause blurred or fluctuating vision and may result in glare or haloes around lights, particularly at night [6]. Dry eye disease also significantly reduces all components of sleep quality even after adjusting for confounders [6]. All these contribute to a reduced quality of life in patients with DED.

Although many studies have elucidated the substantial effect of DED on quality of life, few large studies have evaluated the association between the severity of DED and quality of life. To evaluate associations between DED severity and quality of life among patients with DED, we performed a secondary analysis of rich data from the Dry Eye Assessment and Management (DREAM) study [7], a large-scale, multicenter, placebo-controlled clinical trial of a well-characterized cohort of DED patients.

## 2. Methods

The DREAM Study (ClinicalTrials.gov identifier NCT02128763, registered on 28 April 2014), funded by the National Eye Institute, was designed to evaluate the efficacy and safety of omega-3 fatty acid supplements for treating dry eye disease [7]. The details of the DREAM study design, study procedures for assessment of dry eye symptoms and signs, and the study results were previously reported [4,7,8,9]. Only the features relevant to this paper were described here. The study followed the principles of the Declaration of Helsinki. Institutional review board approval was obtained from each clinical center listed at http://links.lww.com/ICL/A131 (accessed on 15 November 2024). Written informed consent was obtained from all participants.

### 2.1. Participants

The study enrolled a total of 535 adult patients with DED from 27 clinical centers in the United States between October 2014 and July 2016. Participants were randomized in a 2:1 ratio, with the active group receiving an oral omega-3 fatty acid supplement (2000 mg eicosapentaenoic acid [EPA] and 1000 mg docosahexaenoic acid [DHA] per day), and the placebo group receiving refined olive oil.

Major eligibility criteria included age of 18 years or older, experiencing dry eye symptoms for at least 6 months prior to the screening visit, using or intending to use artificial tears at least twice daily for the 2 weeks preceding the screening visit, and having an Ocular Surface Disease Index (OSDI) score between 25 and 80 at the screening visit and 21 to 80 at the baseline visit. Patients also need to meet at least two of the following four criteria for dry eye signs in the same eye at both the screening and baseline visits: (1) conjunctival lissamine green staining score of 1 or more, (2) corneal fluorescein staining score of 4 or more, (3) tear film break-up time (TBUT) of 7 s or fewer, and (4) Schirmer test score of 1 to 7 mm per 5 min.

### 2.2. Evaluation of DED Symptoms and Signs

These DED symptoms and signs were measured at baseline, 6, and 12 months.

The DED symptoms were assessed using the OSDI for the vision-related function, ocular symptoms, and environmental triggers. The total OSDI score and each subscale score range from 0 to 100, with higher scores indicating more severe DED symptoms.

DED signs in each eye were assessed following a standardized protocol. Corneal fluorescein staining was assessed in 5 areas of the cornea using the National Eye Institute with a total possible score of 0 to 15 per eye (a higher score indicating greater abnormality). Conjunctival lissamine green staining was assessed in the nasal and temporal areas for a total possible score of 0 to 6 per eye (a higher score indicating a greater abnormality). The TBUT was measured three times per eye for the time it took for the first break in the tear film to appear, and the average value was calculated for analysis. Lower TBUT values indicated a higher level of abnormality. The Schirmer test was conducted to evaluate tear production, with lower scores indicating greater abnormality. Tear osmolarity measured the concentration of solutes in the tear film on a scale of 275 to 400 mOsm/L, and higher scores indicated greater abnormality. The meibomian gland dysfunction (MGD) was assessed by the clinicians based on the degree of plugging and secretion observed at the lower eyelid margin, with total scores ranging from 0 to 6 (higher scores indicating more severe MGD).

### 2.3. Evaluation of Quality of Life

Quality of life was measured by the Short Form Health Survey (SF-36) version 2.0 [10]. The 36-item questionnaire was divided into two components: the Physical Component Summary (PCS) and Mental Component Summary (MCS), with 8 subscales (physical functioning, role limitations due to physical health, bodily pain, general health, vitality, social functioning, role limitations due to emotional problems, and mental health). The scores of PCS and MCS ranged from 0 to 100, with higher scores indicating a higher level of well-being [10]. The SF-36 questionnaire was administered to each patient at baseline, 6, and 12 months.

### 2.4. Statistical Analysis

Data from both treatment groups were combined for all statistical analysis because the results of the DREAM study showed that there were not any significant differences between participants randomized to n-3 fatty acids or placebo groups in any primary outcome and secondary outcomes [8], and exploratory outcomes [11].

Due to the non-normality of the DED symptoms and signs, the Spearman correlation coefficient (rho) was used to assess the association between the dry eye symptoms, signs, and SF-36 at baseline. The dry eye symptoms were evaluated for the OSDI overall total score and 3 subscale scores (vision-related function, ocular symptoms, and environmental triggers). Dry eye signs in the worse eye (specific to each sign) were analyzed for conjunctival staining score, corneal staining score, Schirmer test score, TBUT, tear osmolarity, and MGD. The SF-36 scores were analyzed for the physical component score and the mental component score separately following the SF-36 user’s manual [12]. Furthermore, partial Spearman correlation coefficients adjusted by randomized treatment group were calculated for evaluating the associations between changes in dry eye symptoms and signs, and change in SF-36 from baseline at 12 months. The 95% confidence intervals (95% CI) for Spearman correlation coefficients and associated *p*-values were calculated using bootstrap [13].

The strength of association was determined based on Spearman correlation coefficients [14]: 0 to 0.19 = very weak, 0.20 to 0.39 = weak, 0.40 to 0.59 = moderate, 0.60 to 0.79 = strong, and 0.80 to 0.99 = very strong. All statistical analyses were performed using SAS version 9.4 (SAS Institute, Cary, NC, USA) and two-sided *p* < 0.05 were considered statistically significant.

## 3. Results

### 3.1. Baseline Characteristics

The baseline characteristics of the 535 study participants with moderate-to-severe DED are summarized in Table 1. The mean ± standard deviation (SD) of age was 58 ± 13.2 years, with most participants being women (81%) and White (74%). The Hispanic or Latino ethnic group consisted of 13% of the participants, while 52 (9.7%) had Sjögren syndrome based on the 2012 American College of Rheumatology classification criteria.

At baseline, the mean ± SD for the total OSDI score was 44.4 ± 14.2. The mean ± SD for each OSDI subscale score was 37.8 ± 17.7 for the vision-related function subscale, 46.6 ± 17.1 for the ocular symptoms subscale, and 55.6 ± 24.5 for the environmental triggers subscale. The mean ± SD for DED signs was 3.3 ± 1.5 for conjunctival staining score, 4.4 ± 3.1 for corneal staining score, 2.7 ± 1.4 for tear break-up time (sec), and 8.2 ± 6.3 for Schirmer’s test (mm in 5 min), 308 ± 1.4 for tear osmolarity, and 1.7 7 ± 0.9 for MGD. To be enrolled in the study, the patients were required to have at least two of the four signs (conjunctival staining score, corneal staining score, tear break-up time, and Schirmer test score). There were 192 (35.9%) patients with two DED signs fulfilling the criteria for enrollment, 203 (37.9%) patients with three signs, and 140 (26.2%) patients with four DED signs. The mean ± SD of the SF-36 score was 47.5 ± 9.7 for the physical component, and 52.3 ± 9.4 for the mental component.

### 3.2. Baseline Association of Dry Eye Symptoms and Signs with SF-36

Baseline correlations between the OSDI and SF-36 are shown in Table 2. More severe DED symptoms (as indicated by higher OSDI total score) were significantly associated with worse quality of life in both the SF-36 physical component score (rho = −0.13, *p* = 0.002) and the mental component score (rho = −0.09, *p* = 0.03). A worse OSDI vision-related function subscale score was significantly associated with a worse SF-36 physical component score (rho = −0.18, *p* < 0.001), while a worse ocular symptoms subscale score was significantly associated with a worse mental component score (rho = −0.15, *p* < 0.001).

Baseline correlations between dry eye signs and SF-36 are summarized in Table 3. A worse SF-36 physical component score was significantly associated with more severe signs in terms of the corneal staining score (rho = −0.22, *p* < 0.001), Schirmer test score (rho = 0.11, *p* = 0.01), tear break-up time (rho = 0.14, *p* < 0.001), and tear osmolarity (rho = −0.12, *p* = 0.02). However, there was no significant correlations between the SF-36 mental component score and dry eye signs (*p* ≥ 0.39).

Table 4 compares SF-36 scores across three groups of patients defined by the number of signs fulfilling the enrollment criteria. The mean SF-36 physical component score significantly decreased with the increase in the number of signs fulfilling the criteria, with mean SF-36 scores of 49.7, 46.6, and 45.7 for patients fulfilling two, three, and four sign criteria, respectively (*p* < 0.0001). However, such a significant association was not observed in the SF-36 mental component score (*p* = 0.87).

### 3.3. Associations of Changes in Dry Eye Symptoms and Signs with Changes in SF-36 from Baseline at 12 Months

Among 486 patients who completed the 12-month follow-up, the mean change from baseline at 12 months was significant for the OSDI overall score (mean change = −11.6, *p* < 0.0001) and each subscale score (−10.4 for vision-related function score, −12.6 for ocular symptom score, −12.7 for environment triggers score, all *p* < 0.0001) and the dry eye signs (−0.8 for conjunctival staining score, −1.1 for corneal staining score, 1.2 for Schirmer test score, 1.4 s for tear break-up time, −4.3 mOsm/L for tear osmolarity, and −0.3 for MGD, all *p* < 0.001). However, there was no significant change in SF-36 physical component score (mean change = 0.1, *p* = 0.80) and mental component score (mean change = −0.3, *p* = 0.40).

Table 5 demonstrates the correlations between changes from baseline in dry eye symptoms and changes from baseline in the SF-36 at 12 months. Only the ocular symptoms subscale was significantly associated with the SF-36 mental component score (rho = −0.09, *p* = 0.04).

Table 6 demonstrates the correlations between changes from baseline in dry eye signs and changes from baseline in SF-36 at 12 months. Change in the Schirmer test score was significantly associated with change in the SF-36 mental component score (rho = 0.09, *p* = 0.04), and change in the tear osmolarity score was significantly associated with change in the SF-36 physical component score (rho = 0.16, *p* = 0.004). There was no other significant association between changes in DED signs and changes in the SF-36.

## 4. Discussion

This study evaluated associations between the severity of DED symptoms and signs and the quality of life in a large, demographically diverse cohort of patients with moderate-to-severe DED. We found a weak yet statistically significant correlation between the severity of DED symptoms and signs with both the physical and mental components of quality of life assessed by the generic SF-36. Dry eye symptoms as measured by OSDI are weakly correlated with both the physical component and mental component of SF-36 (absolute correlation coefficient ≤ 0.13). Similarly, the OSDI vision-related function subscale score is weakly correlated with the SF-36 physical component score (rho = −0.18) and the ocular symptoms subscale is weakly correlated with the SF-36 mental component score (rho = −0.15). However, there is no significant correlation between the subscale of environmental triggers and SF-36. These weak or non-significant correlations may be due to the fact that the SF-36 is a measure of quality of life not specific to dry eye disease. Thus, it is limited in capturing all of the effects of dry eye on the quality of life. These weak correlation findings are consistent with prior studies that found ocular discomfort scores were correlated moderately to strongly with the OSDI, and with measures of activities of daily living. Specifically, Sayegh et al. found a significant correlation between OSDI scores and ocular discomfort (Spearman correlation coefficient, rho = 0.47–0.67) and with measures of activities of daily living (rho = 0.39–0.65) [4]. In another study, Dias et al. investigated the quality of life in patients with primary Sjögren’s and, after matching with healthy controls by age and gender, found that SF-36 and World Health Organization Quality of Life Assessment (WHOQOL-BREF) values were lower in patients with primary Sjögren’s (*p* < 0.001), except in the WHOQOL-BREF environment domain. They also found that pain and fatigue were significant predictors of poorer quality of life [15].

We also found a significant yet weak correlation between four out of the six individual dry eye signs and the physical component score of the SF-36 at baseline. The number of signs fulfilling the enrollment criteria at baseline was significantly associated with the physical component score of the SF-36. This implies that the cumulative burden of DED signs may have a significant influence on the physical health of dry eye patients.

Prior studies have found an association between the presence of DED and mental health. Through meta-analysis, Wan et al. reported a significantly higher prevalence and severity of depression and anxiety in DED patients than in controls [16]. Mertzanis et al. found that all SF-36 subscales except mental health (Effect Size (ES) = 0.12) were lower in the Sjögren syndrome group than in the normal control group. Even the group with severe disease scored lower than the control group across all domains (ES range: −0.14 to −0.91) except role–emotional (ES = 0.13) and mental health (ES = 0.23) [5]. Different from previous studies, our study evaluated associations between the severity of DED (in terms of symptoms and signs) and the mental component of SF-36. However, we only found a weak yet significant association between the OSDI ocular symptoms subscale and the SF-36 mental component score (rho = −0.15). Furthermore, we found the number of signs fulfilling the enrollment criteria at baseline was not significantly associated with the mental component score of the SF-36. These different findings from our study and previous studies could be due to the differences in the study population and the severity of dry eye diseases. The conflicting results suggest that further investigation into the association between mental health and DED is needed.

The impact of DED extends across a wide spectrum of individuals’ quality of life beyond the visual component and encompasses aspects of activities of daily living, social and physical functioning, and workplace productivity [2]. Using data from a large therapeutic trial, Cornec et al. identified the main determinants of health-related quality of life (HRQOL) impairments in patients with active primary Sjögren’s syndrome, with one-third of the patients exhibiting low, moderate, and high systemic activity. They found that lower SF-36 scores indicated a marked HRQOL impairment in the population with active primary Sjögren’s syndrome and were most strongly associated with patient-reported symptoms, and no associations between systemic activity level and HRQOL [17]. Overall, compared to the weak associations we found, prior studies showed a stronger relationship between quality of life and dry eye symptoms and signs. This may be because previous studies included patients with mild DED or controls without DED, while our cohort only included those with moderate-to-severe DED. Nevertheless, our findings add to the literature highlighting the importance of assessing the quality of life in patients with DED because it mirrors the impact of DED on their daily lives.

In the DREAM study, there were significant improvements between baseline and 12 months in DED symptoms and signs, but no significant change in SF-36. We did not find any significant association between the change in dry eye symptoms and the change in SF-36 except for the weak yet significant correlation between the ocular-related symptoms subscale and mental component score (rho = −0.09, *p* = 0.04). Similarly, we only found a weak yet significant association between the change in the Schirmer test score and the change in the mental component score of the SF-36 (rho = 0.09, *p* = 0.04). Although we found the change in osmolarity score was significantly correlated with the change in the physical component score of the SF-36 (rho = 0.16, *p* = 0.004), the correlation was in an unexpected direction (i.e., worsening in tear osmolarity was associated with an improvement in the physical component score).

While numerous investigations have previously investigated the significant influence of DED on quality of life, the scarcity of large-scale studies focusing on the direct association between the severity of DED and quality of life underscores the strength of our study. By conducting a secondary analysis of rich data from the large meticulously characterized cohort of the multicenter DREAM study, we were able to provide a robust assessment of the impact of DED severity on quality of life. Furthermore, this extensive dataset encompasses a comprehensive standardized evaluation of dry eye symptoms and signs. One limitation of our study involves the use of questionnaires. The use of self-reported quality-of-life measures, such as the SF-36, relies on participants’ subjective perceptions and may be influenced by individual biases or social desirability, potentially impacting the validity of the results. Furthermore, the DREAM study only enrolled patients with moderate-to-severe DED without the inclusion of patients with mild DED, which may limit our capability to find a stronger correlation. Future research should explore the quality of life specific to dry eye disease and evaluate the impact of dry eye disease on QOL in dry eye patients with wide range of severity.

In conclusion, our study has shed light on the multifaceted impact of dry eye disease severity on individuals’ lives. The findings have underscored the need for a comprehensive approach to DED management that addresses its physical, psychological, and socioeconomic dimensions. As we move forward, it is imperative that healthcare professionals consider the holistic well-being of DED patients, offering not only symptom relief but also support for the emotional and practical challenges they face in their daily lives. Additionally, further research is warranted to explore the complex interplay between DED and quality of life, particularly in the mental health component, allowing for the development of more targeted interventions and therapies to improve the lives of those affected by this condition.

## Figures and Tables

**Table 1:** Baseline characteristics of patients (n = 535).

Baseline Characteristics	
Age—years, mean (SD)	58 (13.2)
Sex—no. (%)	
Female	434 (81.1)
Male	101 (18.9)
Race—no. (%)	
White	398 (74.4)
Black	64 (12.0)
Other	73 (13.6)
Ethnic Group—no. (%)	
Hispanic or Latino	68 (12.7)
Other	467 (87.3)
Sjögren Syndrome Status *—no. (%)	
Sjögren syndrome	52 (9.7)
Non-Sjögren syndrome	456 (85.2)
Unknown **	27 (5.1)
OSDI Score, mean (SD)	
Total	44.4 (14.2)
Vision-related function subscale	37.8 (17.7)
Ocular symptoms subscale	46.6 (17.1)
Environmental triggers subscale	55.6 (24.5)
Signs of Dry Eye Disease, mean (SD)	
Conjunctival staining score	3.3 (1.5)
Corneal staining score	4.4 (3.1)
Tear break-up time—s	2.7 (1.4)
Result on Schirmer’s test—mm in 5 min	8.2 (6.3)
Osmolarity (mOsm/L)	307.7 (17.5)
Meibomian gland dysfunction	1.7 (0.9)
Composite severity score	0.6 (0.3)
SF-36	
Physical component score	47.5 (9.7)
Mental component score	52.3 (9.4)

* Sjögren syndrome was defined by the 2012 American College of Rheumatology classification criteria for Sjögren syndrome based on serology and ocular surface staining. ** Without serology for antibody test for Sjögren syndrome.

**Table 2:** Correlations between dry eye symptoms and SF-36 at baseline (n = 535 patients).

Dry Eye Symptoms (Higher Is Worse)	SF-36 (Higher Is Better)			
	Physical Component Score		Mental Component Score	
	rho (95% CI)	*p*-Value	rho (95% CI)	*p*-Value
OSDI: total score	−0.13 (−0.21, −0.05)	0.002	−0.09 (−0.18, −0.01)	0.03
Vision-related function subscale	−0.18 (−0.26, −0.09)	<0.001	−0.07 (−0.15, 0.01)	0.10
Ocular symptoms subscale	−0.06 (−0.15, 0.02)	0.15	−0.15 (−0.23, −0.06)	<0.001
Environmental triggers subscale	0.00 (−0.08, 0.09)	0.95	0.00 (−0.08, 0.09)	0.99

**Table 3:** Correlations between dry eye signs and SF-36 at baseline (n = 535 patients).

Dry Eye Signs *	SF-36 (Higher Is Better)			
	Physical Component Score		Mental Component Score	
	rho (95% CI)	*p*-Value	rho (95% CI)	*p*-Value
Conjunctival staining score (lower is better)	0.02 (−0.06, 0.11)	0.60	0.00 (−0.08, 0.09)	0.99
Corneal staining score (lower is better)	−0.22 (−0.30, −0.14)	<0.001	−0.03 (−0.11, 0.06)	0.50
Schirmer test (mm) (higher is better)	0.11 (0.02, 0.19)	0.01	0.04 (−0.05, 0.12)	0.39
Tear break-up time (seconds) (higher is better)	0.14 (0.06, 0.23)	<0.001	0.02 (−0.06, 0.11)	0.56
Osmolarity (mOsm/L) (higher is worse)	−0.12 (−0.21, −0.02)	0.02	−0.02 (−0.12, 0.07)	0.63
Meibomian gland dysfunction (higher is worse)	−0.05 (−0.14, 0.03)	0.22	−0.03 (−0.11, 0.06)	0.54

* The eye with worse signs was used in the calculation.

**Table 4:** Comparison of SF-36 scores across three groups of patients defined by the number of signs fulfilling the criteria at baseline (n = 535 patients).

	Mean (SD)			*p*-Value **
SF-36 Score	2 Signs Fulfilling Criteria * (n = 192 Patients)	3 Signs Fulfilling Criteria * (n = 203 Patients)	4 Signs Fulfilling Criteria * (n = 140 Patients)	
Physical component score	49.74 (9.77)	46.61 (9.52)	45.69 (9.19)	<0.001
Mental component score	52.61 (8.98)	52.12 (9.51)	52.31 (9.80)	0.87

* The enrollment criteria required the presence of at least two of the following four signs in the same eye at both the screening visit and the baseline visit: (1) a conjunctival lissamine green staining score of 1 or more; (2) corneal fluorescein staining score of 4 or more; (3) tear break-up time of 7 s or less; or (4) a result on Schirmer’s test with anesthesia of 1 to 7 mm in 5 min. The grouping of patients is based on the eye with more signs fulfilling the criteria for this person-level comparison of SF-36 scores. ** From analysis of variance.

**Table 5:** Correlations between changes in dry eye signs and change in SF-36 at 12 months from baseline (n = 486 patients).

Change in Dry Eye Symptoms (Month 12—Baseline)	Change in SF-36 (12 Months—Baseline)			
	Physical Component Score		Mental Component Score	
	rho * (95% CI)	*p*-Value	rho * (95% CI)	*p*-Value
OSDI: total score	−0.04 (−0.13, 0.05)	0.41	−0.08 (−0.16, 0.01)	0.09
Vision-related function subscale	−0.04 (−0.13, 0.05)	0.42	−0.09 (−0.17, 0.00)	0.06
Ocular symptoms subscale	0.02 (−0.07, 0.10)	0.74	−0.09 (−0.18, −0.00)	0.04
Environmental triggers subscale	−0.07 (−0.16, 0.02)	0.12	0.01 (−0.08, 0.10)	0.75

* Partial correlation coefficients (rho) are calculated by adjusting for the treatment group.

**Table 6:** Correlations between changes in dry eye symptoms and signs at 12 months from baseline (n = 486 patients).

Change in Dry Eye Signs ** (Month 12—Baseline)	Change in SF-36 (Month 12—Baseline)			
	Physical Component Score		Mental Component Score	
	rho * (95% CI)	*p*-Value	rho * (95% CI)	*p*-Value
Conjunctival staining score (lower is better)	−0.03 (−0.12, 0.05)	0.44	0.01 (−0.08, 0.10)	0.88
Corneal staining score (lower is better)	0.02 (−0.07, 0.11)	0.72	−0.03 (−0.11, 0.06)	0.57
Schirmer test (mm) (higher is better)	−0.01 (−0.10, 0.08)	0.79	0.09 (0.00, 0.18)	0.04
Tear break-up time (seconds) (higher is better)	0.00 (−0.09, 0.00)	0.96	0.02 (−0.07, 0.11)	0.61
Osmolarity (higher is worse)	0.16 (0.05, 0.26)	0.004	−0.09 (−0.19, 0.02)	0.12
Meibomian gland dysfunction (higher is worse)	0.01 (−0.08, 0.10)	0.79	0.04 (−0.05, 0.13)	0.42

* Partial Spearman correlation coefficients (rho) are calculated by adjusting for the treatment group. ** The change (Month 12—baseline) from the eye with worse signs at baseline was used for analysis.

## Data Availability

All the data used for this study are available at https://hyperprod.cceb.med.upenn.edu/dream_download/index.php.
